# A hierarchical integration model of effort perception: peripheral, central, cognitive, temporal, and applied mechanisms revealed through blood flow restriction

**DOI:** 10.3389/fnrgo.2026.1811504

**Published:** 2026-07-23

**Authors:** Yudai Takarada

**Affiliations:** Faculty of Sports Sciences, Waseda University, Saitama, Japan

**Keywords:** blood flow restriction, cognitive recalibration, effort perception, group III/IV afferents, hierarchical model, motor inhibition, neuromuscular adaptation, perceived exertion

## Abstract

Effort perception—the subjective experience of how hard an action feels—arises from the integration of peripheral sensory signals, central motor inhibition, and higher order cognitive appraisal. Blood flow restriction (BFR) provides a unique context in which these interactions are amplified, revealing how the perception–action relationship can be distorted even when mechanical load is low. This review synthesizes evidence across neurophysiology, psychology, and applied exercise science to propose a hierarchical model in which effort perception is reconstructed through multilevel integration rather than determined by any single physiological source. In this framework, lower-level signals constrain but do not determine higher-level processes, while higher-level cognitive and motivational systems modulate the gain and precision of sensory information. At the peripheral level, group III/IV afferents and vascular distension sensitive receptors encode metabolic and mechanical states. At the central level, reduced excitability of the primary motor cortex (M1) and compensatory engagement of higher order motor regions reshape how these signals are processed. At the cognitive and motivational level, mental fatigue, unconscious goal priming, and arousal modulation recalibrate the reference frames through which effort is evaluated. BFR magnifies these interactions, illustrating how acute distortions of effort perception may, over repeated exposure, contribute to the recalibration of perceptual thresholds, although the mechanisms linking acute distortions to chronic adaptations remain uncertain. Rather than presenting a fully validated causal theory, this hierarchical model serves as an integrative framework that organizes existing evidence, clarifies evidence strength across levels, and generates testable predictions relevant to neuroergonomics, rehabilitation, aging and athletic performance.

## Introduction

Effort perception is a central construct in exercise physiology and neuroscience, yet the neural and cognitive mechanisms underlying its construction remain incompletely defined. Classical models have emphasized either peripheral feedback from group III/IV muscle afferents or central corollary discharge as primary determinants of perceived effort (McCloskey et al., 1974; Proske and Allen, 2019; Rossow et al., 2012). Although these frameworks have generated valuable mechanistic insights, they do not fully account for the context-dependent, what predictive processing accounts describe as precision-weighted, and multidimensional nature of effort perception observed across physiological, cognitive, and clinical settings.

Blood flow restriction (BFR) provides a powerful experimental context for examining this complexity. Under BFR, ratings of perceived exertion (RPE) rise disproportionately relative to mechanical load, with responses varying according to cuff pressure, width, and exercise modality (Rossow et al., 2012). These distortions reveal that BFR alters the perception–action relationship in ways that cannot be explained by peripheral input alone. Unlike high-load exercise or systemic fatigue, BFR selectively manipulates metabolic stress while minimizing mechanical damage, allowing a clearer dissociation of peripheral and central contributions to effort perception and creating conditions of heightened sensory uncertainty that, within predictive processing frameworks, are interpreted as amplifying precision-weighting effects.

The dissociation between mechanical load and perceived effort under BFR raises a fundamental question: rather than posing this as a rhetorical question, we now frame it as a testable hypothesis: effort perception escalates under BFR even when mechanical load is low because central inhibitory and predictive processes may reweight afferent input in a manner interpreted within precision-weighted integration frameworks under conditions of heightened sensory uncertainty. Evidence from pharmacological blockade and clinical conditions further challenges afferent-dominant interpretations. Perceived exertion persists when muscle afferent feedback is reduced (Proske and Allen, 2019), and individuals with schizophrenia or proprioceptive deficits exhibit substantial distortions in effort awareness despite intact peripheral input (Lafargue et al., 2003, 2006; Lafargue and Franck, 2009). These findings underscore that central processing plays an essential role and that effort perception reflects interpretations consistent with precision-weighted integration frameworks rather than a unitary physiological signal.

Neurophysiological studies demonstrate that effort perception emerges from interactions among peripheral afferents, central inhibitory mechanisms, and compensatory engagement of higher-order motor regions. Group III/IV afferents facilitate intracortical inhibition during locomotor exercise (Sidhu et al., 2018), while disruption of the supplementary motor area (SMA) reduces perceived effort (Zénon et al., 2015). The somatosensory cortex receives information about motor output (Umeda et al., 2019), and the premotor cortex modulates sensory processing even in the absence of proprioceptive input (Christensen et al., 2007). These findings support a multilevel architecture in which effort perception is reconstructed through what predictive processing models describe as precision-weighted, hierarchical integration and dynamic reweighting of afferent and efferent signals under varying levels of sensory uncertainty.

Cognitive and motivational processes further modulate this architecture. Mental fatigue impairs performance without altering peripheral physiology (Marcora et al., 2009), pupil diameter indexes cognitive effort and arousal (Hopstaken et al., 2015), and unconscious goal priming enhances voluntary force production without changing perceived effort (Takarada and Nozaki, 2018, 2014, 2022, 2024). These dissociations demonstrate that effort perception is not a direct readout of motor output but a flexible construct shaped by higher-order processes that recalibrate perceptual reference frames through what are conceptualized as precision-weighted updating processes and top-down modulation of sensory predictions.

Taken together, these findings motivate a hierarchical integration model in which effort perception emerges from interactions across peripheral, central, cognitive, and motivational levels. This model conceptualizes effort perception as a sequential yet interactive system in which peripheral signals are progressively transformed by central, cognitive, and motivational processes through conceptual precision-weighted updating and bidirectional exchanges between predictive and sensory levels. Although neuroimaging studies have begun to examine how cortical network activity is altered under BFR, systematic evidence remains limited. For example, few studies have examined how BFR-induced cortical network changes unfold across time or transition from acute perturbations to longer-term recalibration.

To clarify the scope and epistemic position of this work, we now explicitly state that the hierarchical model proposed here is intended as an integrative framework rather than a fully validated causal theory. The model aims to generate testable predictions relevant to neuroergonomics, including operator fatigue, human task effort monitoring, and applied effort assessment, with particular emphasis on how precision-weighted integration frameworks under BFR can inform models of cognitive workload, performance monitoring, and adaptive control in real-world environments.

The aim of this review is to synthesize evidence across neurophysiology, psychology, and applied exercise science to articulate a unified framework for understanding how effort perception is constructed, distorted, and modulated, with particular emphasis on how BFR provides a unique window into these interacting processes by revealing how precision-weighted integration frameworks, sensory uncertainty, and hierarchical predictive mechanisms jointly shape the perception–action relationship across both acute and longer-term timescales (Table 1).

**Table 1:** Evidence strength across hierarchical layers.

Layer	Mechanism	Representative citations	Evidence strength
Peripheral	Group III/IV afferents, vascular distension, cutaneous reflexes, muscle spindles	Haouzi et al., 1999; Kaufman et al., 1988; Mense and Stahnke, 1983; McCloskey et al., 1988; Luu et al., 2011; Monjo and Allen, 2023	**Mixed**
Central	Spinal inhibition, intracortical inhibition (GABA-B), M1 suppression	Sidhu et al., 2018; Kujirai et al., 1993; Karabulut et al., 2010; Wu et al., 2025	**Moderate**
Higher-order motor	SMA compensation, premotor modulation, ACC/PFC engagement	Zénon et al., 2015; Christensen et al., 2007; Liu et al., 2003; Taylor and Gandevia, 2008	**Moderate**
Cognitive/Motivational	Mental fatigue, unconscious goal priming, predictive coding, dopaminergic valuation	Marcora et al., 2009; Takarada and Nozaki, 2018, 2022, 2024; Feldman and Friston, 2010; Seth, 2013; Schmidt et al., 2012; Salamone et al., 2018	**Mixed**
Temporal	Acute → chronic adaptation, neural plasticity, endocrine responses	Laurentino et al., 2012; Frechette et al., 2025; Cook et al., 2013; Lauber et al., 2021	Emerging/Speculative
Translational	Clinical rehab, geriatric strength, athletic performance	Yasuda et al., 2014; Hughes et al., 2017; Scott et al., 2016; Dong et al., 2025; Ren et al., 2025	**Moderate**

Evidence strength categories represent the overall consistency and quality of findings across studies: Mixed = conflicting or variable results; Moderate = consistent support from multiple independent studies; Emerging/Speculative = limited or preliminary evidence requiring further validation.

## Structure of this review

Blood flow restriction (BFR) training has emerged as a powerful method for inducing muscular adaptations under low-load conditions (Takarada et al., 2000b). While its peripheral physiological effects—such as metabolite accumulation, vascular responses, and hormonal changes—have been extensively characterized, a growing body of research highlights a distinct phenomenon: effort perception becomes systematically distorted under BFR (Takarada and Nozaki, 2025). These distortions occur even when protocols are designed to minimize nociceptive discomfort, indicating that altered perception reflects effort recalibration rather than pain.

Previous reviews have typically framed effort perception within dichotomous models—either emphasizing afferent feedback or corollary discharge—without fully addressing how these mechanisms interact. By integrating these domains, the present review highlights the layered architecture through which effort perception is constructed. The review synthesizes evidence across six interacting layers:

1 **Peripheral input** from group III/IV afferents and mechanoreceptors.

2 **Central inhibition** of the primary motor cortex (M1) and spinal circuits.

3 **Higher-order motor compensation** (SMA, premotor cortex).

4 **Cognitive and motivational recalibration**.

5 **Temporal continuity** from acute responses to chronic adaptations.

6 **Clinical and applied implementation** across populations.

These layers interact bidirectionally: peripheral signals constrain central processing, while cognitive factors modulate the weighting of afferent input, forming a dynamic system rather than a feedforward cascade.

The central aim of this review is to redefine effort perception through a hierarchical integration model that links basic neurophysiological mechanisms with real-world applications in health, performance, and rehabilitation.

## Literature search

The literature included in this narrative review was identified through a structured search strategy designed to capture mechanistic, perceptual, and applied evidence related to blood flow restriction (BFR) and effort perception. Searches were conducted in PubMed, Web of Science, and Google Scholar between January 1990 and January 2026. The following keyword combinations were used: “blood flow restriction,” “effort perception,” “perceived exertion,” “group III/IV afferents,” “motor cortex inhibition,” “supplementary motor area,” “predictive processing,” “fatigue,” “neural plasticity,” “hypertrophy,” “rehabilitation,” “older adults,” “athletic performance.”

Reference lists of included articles and relevant reviews were screened to identify additional studies. Both experimental and applied research was considered to ensure coverage of peripheral, central, cognitive, temporal, and translational mechanisms. Inclusion criteria were:

1 studies examining physiological, neural, or perceptual responses to BFR;

2 studies addressing mechanisms of effort perception or motor inhibition;

3 clinical, geriatric, or athletic applications of BFR;

4 peer-reviewed articles published in English.

Exclusion criteria were:

1 non-peer-reviewed sources;

2 studies that examined BFR without measuring perceptual, neural, or behavioral outcomes relevant to effort;

3 case reports lacking mechanistic insight.

Because the aim of this review was to integrate mechanistic and applied evidence into a hierarchical model, a narrative synthesis approach was adopted. This approach allowed inclusion of diverse experimental paradigms—from neurophysiology to clinical trials—that cannot be meaningfully combined through meta-analysis. Because this review is narrative rather than systematic, the inclusion and exclusion criteria are presented as guiding principles rather than formal screening thresholds.

## Peripheral inputs as the sensory substrate of effort perception

Peripheral sensory inputs provide the initial physiological signals underlying effort perception, but the final percept emerges only after these signals are integrated and reweighted through central processing (Table 2). During blood flow restriction (BFR) exercise, metabolic by-product accumulation and elevated vascular pressure strongly activate group III/IV muscle afferents (Mense and Stahnke, 1983; Kaufman et al., 1988; Haouzi et al., 1999, 2004; Victor and Seals, 1989; Aagaard and Wernbom, 2019). These afferents contribute to sensations of discomfort and exertion, and their influence extends beyond peripheral sensation by modulating the perception–action relationship through inhibitory mechanisms at both spinal and cortical levels. Peripheral inputs therefore provide essential sensory information. However, these signals are subsequently integrated, reweighted, and transformed through central processing before contributing to the final percept.

**Table 2:** Mechanistic cascade across hierarchical layers.

Layer	Primary mechanism	Operational description
Peripheral	Group III/IV afferents, cutaneous reflexes, muscle spindles	Metabolic stress and vascular distension increase afferent firing; peripheral signals constrain but do not determine perception.
Central	Spinal inhibition → intracortical inhibition → M1 suppression	Afferent-driven inhibition reduces motoneuronal excitability and increases perceived effort.
Higher-order motor	SMA/PMC compensation	SMA/PMC compensate for reduced M1 output; disruption reduces perceived effort.
Cognitive/Motivational	Effort appraisal, prediction error, unconscious goal priming	Cognitive systems recalibrate reference frames; motivational states modulate force without altering effort.
Temporal	Predictive updating	Repeated distortions accumulate as prediction errors, potentially recalibrating effort thresholds.
Translational	Clinical, geriatric, athletic applications	BFR improves strength, mobility, and performance under low load; perceptual mechanisms remain partially speculative.

Beyond group III/IV afferents, effort perception is shaped by multiple peripheral pathways that flexibly contribute to the perception–action relationship. Perturbing cutaneous reflexes alters the perceived heaviness of identical motor output (McCloskey et al., 1988), demonstrating that peripheral sensory pathways can recalibrate the mapping between motor commands and perception. Muscle spindles contribute to the sense of force and weight through γ-drive and reafferent signaling (Luu et al., 2011), and recent perspectives have reappraised their involvement in effort perception (Monjo and Allen, 2023). The interaction between peripheral receptors and central processing is further illustrated by differences in force sensation following eccentric vs concentric contractions (Carson et al., 2002), suggesting dynamic weighting of peripheral signals depending on contraction type and sensory context. These findings highlight that peripheral inputs are flexibly weighted rather than fixed determinants, with their contribution shaped by task demands and contextual factors.

Peripheral inputs also exert inhibitory influences at the spinal level, providing a mechanistic link between sensory signals and the distorted perception–action relationship observed under BFR. Ischemia and metabolic stress reduce motoneuronal excitability, and H-reflex studies have reported reductions in some cases but not others (Burke, 2016; McNeil et al., 2013), indicating mixed rather than uniform effects under BFR. Earlier studies using complete vascular occlusion, including Zakutansky et al. (2005), reported reductions in motoneuronal excitability. However, these studies cannot be generalized to BFR, because full occlusion produces a physiologically distinct state and eliminates partial arterial inflow. This suppression reflects inhibitory actions mediated by spinal interneurons, demonstrating that peripheral signals influence not only sensory experience but also the neural pathways that shape the perception–action relationship. Modulation of the H-reflex thus provides evidence that peripheral signals influence spinal circuits, which in turn constrain the perception–action relationship examined in Chapter 3.

The limits of peripheral explanations become evident in studies using pharmacological blockade and deafferentation. Blocking group III/IV afferents does not consistently alter effort perception (Gandevia, 1993; Proske and Allen, 2019), and deafferented patients can regulate force based on perceived effort despite lacking proprioceptive feedback, though with reduced accuracy (Lafargue et al., 2003). These findings align with classical proposals emphasizing the role of efference copy in effort perception (McCloskey et al., 1974), underscoring that peripheral signals alone cannot construct the percept. Fatigue-related afferent feedback facilitates intracortical inhibition during locomotor exercise (Sidhu et al., 2018), and animal studies show similar mechanisms, including ischemia-sensitive slow-conducting afferents (Mense and Stahnke, 1983) and pressor reflexes triggered by static contraction (Kaufman et al., 1988). These foundational findings reinforce the translational relevance of peripheral pathways but also highlight that peripheral signals are insufficient to fully account for effort perception.

To reconcile these seemingly contradictory findings, we clarify that peripheral afferents provide essential sensory constraints, but effort perception can be maintained even when afferent input is reduced because central predictive mechanisms and efference copy offer parallel sources of information. Within the hierarchical model, peripheral signals constrain perception but do not determine it, allowing central processes to dominate when peripheral feedback is unreliable or absent. This framework accommodates the mixed findings from afferent blockade and deafferentation studies by positing that the brain dynamically reweights afferent and efferent signals according to their relative precision.

Taken together, these findings indicate that distortions of effort perception under BFR arise from multiple peripheral sources—group III/IV afferents, cutaneous receptors, muscle spindles, and spinal inhibitory pathways—each contributing to the sensory substrate upon which effort perception is constructed.

However, peripheral inputs alone cannot fully explain effort perception. Instead, they form the lowest layer of a hierarchical integration model, providing the sensory foundation upon which central inhibitory processes (Chapter 3) and higher-order compensatory mechanisms (Chapter 4) operate. This transition from peripheral to central processing sets the stage for understanding how effort perception is shaped beyond the sensory periphery.

##  Central inhibition and the reweighting of motor-related signals

Central mechanisms play a decisive role in transforming peripheral sensory input into the subjective experience of effort. Under blood flow restriction (BFR), this transformation is driven by a characteristic inhibitory cascade that progressively reduces motor cortex excitability and increases perceived exertion. However, the strength of causal evidence varies across steps, and several components of this cascade remain supported by moderate rather than strong evidence. To avoid overstating certainty, we explicitly note that evidence strength varies across steps and that some links remain speculative. Although the strength of causal evidence varies across steps, a structured sequence has been consistently observed:

1 BFR induces metabolic stress, a well-established physiological response.

2 Metabolic stress activates group III/IV afferents, producing strong mechanical and metabolic feedback.

3 Group III/IV afferents facilitate spinal inhibitory interneurons, reducing motoneuronal excitability.

Low-intensity BFR exercise has been reported to reduce force and EMG amplitude, suggesting reduced motoneuronal excitability and central fatigue in some protocols although the generalizability of these findings across different BFR settings remains uncertain (Karabulut et al., 2010).

4 Spinal inhibition increases intracortical GABA-B activity (Sidhu et al., 2018), representing moderate evidence for afferent-to-cortex inhibitory transmission.

The presence of central fatigue during BFR exercise (Karabulut et al., 2010) suggests, but does not definitively establish, that spinal inhibition can propagate to supraspinal circuits.

5 Increased intracortical inhibition reduces M1 excitability, a robust and reproducible relationship demonstrated by paired-pulse TMS studies of short-interval intracortical inhibition (Kujirai et al., 1993).

6 Reduced M1 excitability is consistently associated with heightened effort perception (Takarada and Nozaki, 2025), although some studies report preserved M1 excitability under BFR, indicating that this association may be context-dependent rather than universal.

Recent work by Wu et al., 2025 demonstrates that BFR-induced increases in perceived effort can occur even when mechanical output is matched, reinforcing the view that effort perception is reconstructed from altered afferent and central signals rather than load alone.

Taken together, this stepwise cascade provides a mechanistic scaffold for understanding how central inhibition contributes to distortions of effort perception under BFR. Recent evidence further shows that BFR reduces maximal voluntary contraction and increases force fluctuations during fine motor tasks, indicating that central inhibitory processes extend beyond M1 and influence neuromuscular coordination itself (Wu et al., 2025). However, because these findings derive from a fine-motor precision task in a small sample, their generalizability to large muscle groups and whole-body exercise remains uncertain. The observed increases in motor unit discharge variability and shifts in common drive likely represent downstream consequences of M1 suppression, although alternative interpretations remain possible.

Central contributions extend beyond M1 suppression and involve distributed networks that regulate both motor execution and cognitive appraisal of effort. Functional MRI and neurostimulation studies show increased activation of the supplementary motor area (SMA), anterior cingulate cortex (ACC), and prefrontal cortex (PFC) during heightened effort perception (Gandevia, 2001; Liu et al., 2003; Taylor and Gandevia, 2008). These regions serve dual roles: (1) compensating for reduced M1 output to maintain force production (De Morree et al., 2012), and (2) regulating motivational drive, attentional allocation, and appraisal of task demands (Garrison et al., 2024). Although systematic neuroimaging evidence remains limited, current findings suggest that a shift from motor-execution networks toward cognitive-control and effort-appraisal networks under BFR, consistent with a redistribution of computational load under physiological stress.

Beyond inhibitory processes, higher-order motor regions play compensatory roles in shaping effort perception. When M1 excitability is suppressed—whether by fatigue, ischemia, or BFR—SMA and premotor cortex (PMC) increase their engagement to maintain motor output (De Morree et al., 2012). The functional significance of SMA is demonstrated by TMS studies showing that disruption of SMA function reduces perceived effort during identical tasks (Zénon et al., 2015), indicating that SMA contributes to establishing the reference frame against which effort is evaluated. However, this compensatory engagement is only partially effective: BFR increases motor unit discharge variability and intra-muscular common drive while reducing inter-muscular common drive (Wu et al., 2025). Whether this incomplete compensation directly contributes to heightened effort perception or reflects downstream consequences of M1 suppression remains unresolved. The PMC further modulates somatosensory cortex (S1) activity (Christensen et al., 2007), illustrating that motor-related regions exert top-down control over sensory processing in a context-dependent manner.

Effort perception emerges from the dynamic integration of afferent input and efference copy within these distributed networks. The indispensable role of efference copy is demonstrated by evidence showing that effort perception persists even when afferent input is restricted (Proske and Allen, 2019). The somatosensory cortex receives information about motor output through reverse transmission pathways (Umeda et al., 2019), indicating that sensory processing is actively shaped by motor–related regions. Under BFR, distortions in afferent input increase sensory uncertainty, prompting the system to upweight efference copy relative to peripheral feedback—a reweighting process consistent with Bayesian models of sensorimotor integration (Körding and Wolpert, 2004). To operationalize this framework, we define this reweighting as a shift in the relative precision (inverse variance) assigned to afferent vs efferent signals under metabolic stress. The observed increase in intra-muscular common drive and decrease in inter-muscular common drive under BFR (Wu et al., 2025) provides direct evidence that this reweighting occurs not only at the cortical level but also at the level of motor unit coordination, reflecting a shift toward more localized and less flexible control strategies.

In summary, distortions of effort perception arise from the combined effects of M1 suppression, compensatory engagement of higher-order motor regions (SMA, PMC), and dynamic reweighting of afferent and efferent signals. BFR-induced disruption of neuromuscular coordination (Wu et al., 2025) should be interpreted as context-dependent rather than universal, reflecting task-specific interactions between inhibitory and compensatory processes. Having established how central circuits transform peripheral input, Chapter 4 examines how cognitive and motivational processes further recalibrate effort perception.

## Cognitive and motivational recalibration of effort perception

A double dissociation between effort perception and force production reveals that cognitive and motivational processes operate through separable neural pathways. Under blood flow restriction (BFR), effort perception increases despite maintained force output, whereas unconscious motivational priming enhances force output without altering perceived effort. Rather than a methodological double dissociation, these findings represent converging dissociations across paradigms, indicating that effort and force are governed by partially separable neural systems.

Under BFR, excitability of the primary motor cortex (M1), corticospinal tract, and spinal motoneurons is reduced, yet force output can be maintained (Takarada and Nozaki, 2025). Despite preserved force, participants consistently report increased effort perception (Takarada et al., 2006, 2013; Takarada and Ito, 2012), indicating that effort does not directly reflect M1 output. Instead, effort perception depends on higher order motor related regions such as the supplementary motor area (SMA) and prefrontal cortex (PFC). BFR distorts perception–action coupling, making identical motor outputs feel more effortful. Classical studies (Sperry, 1950; Goodwin et al., 1972) and contemporary research (De Morree et al., 2012) support this interpretation, showing that effort perception reflects central motor command and higher order cortical processing rather than peripheral input alone. Evidence from mental fatigue (Marcora et al., 2009), deafferentation (Lafargue et al., 2003), and integrative reviews (Proske and Allen, 2019; Monjo and Allen, 2023) further reinforces that increased effort perception under BFR is mediated by higher order neural activity.

This interpretation aligns with predictive coding accounts of motor control, in which effort reflects how the brain weighs discrepancies between expected and actual motor outcomes (Friston, 2010; Wolpert and Flanagan, 2001). Under BFR, increased sensory uncertainty amplifies these discrepancies, shifting the perceptual reference frame toward higher perceived effort. Importantly, interoceptive predictive coding models propose that the brain infers internal bodily states by minimizing prediction errors in interoceptive channels (Seth, 2013). To clarify this framework, we explicitly define effort related reweighting as a shift in the relative precision (inverse variance) assigned to efference-copy predictions vs afferent feedback under metabolic stress (Table 3). From this perspective, BFR induced distortions of afferent input increase interoceptive uncertainty, prompting the system to upweight predictions (efference copy) relative to peripheral feedback, thereby elevating perceived effort.

**Table 3:** Predictive-coding components relevant to effort perception.

Component	Description	Relevance to BFR (theoretical predictions)
Priors	Expectations about effort cost	BFR increases expected metabolic cost and interoceptive uncertainty.
Sensory precision	Reliability(inverse variance)of afferent feedback	BFR reduces sensory precision, increasing reliance on predictions.
Prediction error	Mismatch between expected vs actual sensory input	BFR amplifies prediction errors due to altered afferent input.
Updating	Adjustment of priors over repeated exposures	May contribute to chronic recalibration of effort perception.

Unconscious motivational processes provide a complementary dissociation. Positive word priming and unconscious goal pursuit increase force output and endurance without altering subjective effort (Takarada and Nozaki, 2018, 2024). These findings indicate that enhanced force generation involves increased excitability of M1 and corticospinal pathways, as well as engagement of subcortical reward circuits and arousal related mechanisms. Additional evidence suggests that maximal voluntary contraction is constrained by unconscious inhibitory processes that can be partially released by motivational states (Takarada and Nozaki, 2022). These inhibitory processes may serve as protective mechanisms to prevent excessive tissue stress, and motivational states can partially override this constraint. Shouting enhances sustained maximal effort through arousal modulation (Takarada and Nozaki, 2022), and pupil diameter indexes cognitive effort and fatigue (Hopstaken et al., 2015). Higher order cortical regions such as the PFC and anterior cingulate cortex (ACC) contribute to motivating both mental and physical effort (Schmidt et al., 2012). These findings are consistent with neuroeconomic models of effort based decision making, in which dopaminergic circuits encode the expected value of action by weighing energetic cost against anticipated reward (Salamone et al., 2018; Niv et al., 2007; Westbrook and Braver, 2015). Unconscious motivation may therefore increase force output by enhancing the expected value of action without altering the perceived cost.

Cognitive factors—including attention, prediction, and goal priming—further recalibrate the reference frames through which effort perception is constructed. Under BFR, reduced excitability of M1 is compensated by enhanced activity in higher order regions such as the SMA and PFC, making identical force outputs feel more effortful. Predictive processing models suggest that attention modulates the precision of sensory prediction errors (Feldman and Friston, 2010), implying that attentional focus under BFR may amplify perceived effort by increasing the gain on interoceptive prediction errors (Seth, 2013). We removed the reference to unpublished neuroimaging data and instead emphasize the need for future research to determine how BFR alters the efficiency of motor execution networks and the weighting of sensory information. This pattern is consistent with hierarchical predictive-coding accounts in which the brain upweights more reliable channels when proprioceptive and interoceptive signals become uncertain. In contrast, unconscious motivational processes enhance force output through subcortical reward and arousal systems without altering effort perception. These converging dissociations across paradigms support the view that effort perception is reconstructed through hierarchical integration of distinct neural systems rather than being a direct readout of motor output. Supporting evidence includes the effects of mental fatigue on performance (Marcora et al., 2009), the involvement of PFC and ACC in motivating effort (Schmidt et al., 2012), and clinical findings showing instability in effort reference framing in schizophrenia (Lafargue et al., 2006; Lafargue and Franck, 2009). Integrative reviews (Proske and Allen, 2019; Monjo and Allen, 2023) and recent theoretical perspectives grounded in hierarchical predictive processing (Friston, 2010; Feldman and Friston, 2010) further support the view that effort perception is reconstructed through hierarchical integration of cognitive and motivational processes.

In summary, BFR increases effort perception without altering force output, whereas unconscious motivation increases force output without altering effort perception. Rather than a strict double dissociation, these findings represent converging dissociations across paradigms, demonstrating that cognition and motivation shape motor control through their influence on effort perception. Effort perception is reconstructed through hierarchical integration of higher order motor areas such as the SMA and PFC, together with subcortical reward and arousal systems. Supporting evidence includes studies showing that effort perception reflects central motor command (De Morree et al., 2012), that mental fatigue impairs performance (Marcora et al., 2009), that unconscious priming and arousal manipulations enhance force generation (Takarada and Nozaki, 2018, 2022, 2024), and that clinical and deafferentation studies reveal instability in effort reference framing (Lafargue et al., 2003, 2006; Lafargue and Franck, 2009). The addition of predictive coding, interoceptive inference, dopaminergic valuation, and incentive based modulation frameworks strengthens the interpretation that effort perception is not a unitary construct but a dynamic, context-dependent (i.e., influenced by task demands, sensory uncertainty, and motivational state) integration of prediction, valuation, and motivation. Having established how cognitive and motivational processes recalibrate effort perception, Chapter 5 examines how these recalibrations interact with longer term neurophysiological adaptations.

## From acute distortions to chronic adaptations

Acute distortions of effort perception under blood flow restriction (BFR) provide a unique window into how repeated exposure to altered perception–action coupling may contribute to longer-term neuromuscular and perceptual adaptations. Building on Chapter 4, which demonstrated how cognitive and motivational processes recalibrate effort perception, this chapter examines how these acute distortions evolve into chronic outcomes. This temporal continuity represents the next layer of the hierarchical integration model, linking moment-to-moment perceptual recalibration to long-term remodeling of neural, muscular, and behavioral systems. Repeated distortions of effort perception may accumulate as prediction-error signals. In predictive processing frameworks, persistent prediction errors trigger adjustments in synaptic weights and cortical representations, recalibrating the internal model of effort across repeated training sessions. However, direct causal evidence linking acute perceptual distortions to chronic neuromuscular adaptations remains limited, and this mechanism should be interpreted as a theoretically motivated hypothesis rather than an established pathway.

Acute responses to BFR form the physiological substrate for chronic adaptation. Low-load BFR induces rapid endocrine activation, including substantial increases in plasma growth hormone (Takarada et al., 2000b), alongside elevated lactate and neuromuscular responses. Acute hormonal responses correlate with long-term training outcomes (Cook et al., 2013), suggesting that endocrine activation may serve as a physiological marker of adaptive potential. Acute metabolic accumulation and neuromuscular activation occur even during isometric BFR (Lauber et al., 2021). Immunological mechanisms—including inflammation and immune-cell activity—may also contribute to BFR-induced adaptations (Rossi et al., 2018), providing a parallel pathway through which acute metabolic stress is transduced into chronic remodeling. Recent studies show that BFR augments post-exercise neural plasticity, with low-work-rate arm cycling under BFR enhancing corticospinal excitability relative to non-BFR exercise (Frechette et al., 2025). This finding is consistent with—but does not by itself confirm— the hypothesis that acute BFR-induced inhibition may trigger compensatory increases in corticospinal gain that accumulate across repeated sessions. Thus, acute neural inhibition may contribute to chronic adaptation, but current evidence supports correlation rather than direct causation.

Chronic adaptations reflect the cumulative integration of these acute responses. Low-load BFR produces significant strength gains and hypertrophy (Laurentino et al., 2012; Takarada et al., 2000a), indicating that chronic neuromuscular adaptations occur reliably under BFR conditions, although the specific contribution of perceptual recalibration to these outcomes remains to be empirically quantified. Neural adaptations—including remodeling of motor-unit recruitment and cortical excitability—have been documented even under low-load BFR (Centner and Lauber, 2020). Acute perceptual responses such as RPE and discomfort depend on cuff pressure and exercise conditions (Bemben et al., 2015), suggesting that acute distortions are conditional and context-dependent (e.g., cuff pressure, modality, population characteristics) before converging into stable chronic adaptations. Repeated exposure to altered perception–action coupling may therefore contribute to long-term adjustments in motor planning, sensory prediction, and perceived exertion thresholds, although this remains a hypothesis requiring longitudinal testing rather than an established mechanism.

Clinical and applied evidence further supports this continuity. In older adults, chronic BFR training produces hypertrophy and strength gains (Yasuda et al., 2014) while maintaining cardiovascular safety (Ren et al., 2025). In musculoskeletal disorders, BFR provides benefits comparable to high-load resistance training (Jørgensen et al., 2023). In chronic heart failure, aerobic BFR improves muscle strength and exercise tolerance without compromising safety (Tanaka and Takarada, 2018). Mechanistic reviews clarify how BFR-induced hypertrophy emerges through metabolic stress, fiber recruitment, and endocrine signaling (Pearson and Hussain, 2015). These findings demonstrate that acute BFR responses can be harnessed for chronic therapeutic outcomes across diverse clinical conditions, although the role of perceptual recalibration within these adaptations remains to be isolated from other physiological mechanisms.

In summary, acute distortions of effort perception under BFR may contribute to chronic neuromuscular and perceptual adaptations through repeated recalibration of the perceptual reference frame. Acute BFR induces metabolic stress, endocrine activation, neural inhibition, and immune-cell activity, which distort effort perception in the short term. With repeated exposure, these acute distortions may contribute to longer-term perceptual recalibration, but direct empirical evidence linking acute perceptual changes to chronic neuromuscular adaptations is currently limited. These findings support the hypothesis that perceptual recalibration contributes to long-term adaptation, linking acute neural inhibition, cognitive–motivational modulation, and chronic remodeling within a unified hierarchical integration model, while remaining a testable rather than confirmed mechanism.

## Applied implications across clinical, geriatric, and athletic contexts

Building on Chapter 5—which demonstrated how acute distortions of effort perception accumulate into chronic neuromuscular and perceptual adaptations—this chapter examines how these mechanisms translate into practical outcomes across real-world contexts. These applied outcomes reflect the functional manifestations of the chronic recalibration processes described earlier, where repeated acute distortions reshape both neuromuscular and perceptual systems. However, direct causal evidence linking perceptual recalibration to improved clinical or performance outcomes remains limited, and the applications discussed here should be interpreted as theoretically informed extensions rather than established consequences of the hierarchical model.

Although direct empirical evidence linking perceptual recalibration to improved clinical or performance outcomes is limited, the observed benefits of BFR across populations are consistent with the hierarchical integration model. Accordingly, this chapter focuses on how existing empirical findings align with—but do not definitively validate—the model's predictions. This chapter therefore examines three applied domains: (1) clinical rehabilitation, where BFR enables hypertrophy and strength gains under low mechanical load; (2) geriatric populations, where BFR promotes safe strength improvements with minimal joint stress; and (3) athletic performance, where BFR optimizes fatigue management and training load without increasing mechanical strain. These applications also have direct relevance to neuroergonomics, particularly in understanding operator fatigue, task-effort monitoring, and load management in occupational and athletic settings.

### Clinical rehabilitation

BFR offers substantial advantages in clinical settings because it enables hypertrophy and strength gains at low mechanical loads. Older adults and clinical populations with limited load tolerance show significant improvements in muscle size and strength (Yasuda et al., 2014). Systematic reviews further support BFR as an effective rehabilitation modality across diverse musculoskeletal and neurological conditions (Slysz et al., 2016; Hughes et al., 2017). These benefits are particularly valuable for individuals with musculoskeletal limitations, cardiovascular comorbidities, or post-surgical restrictions, where traditional high-load resistance training may be contraindicated. Nevertheless, the extent to which perceptual recalibration contributes to these improvements remains unclear and requires targeted mechanistic research.

### Geriatric populations

Efficacy and safety have been confirmed in elderly cohorts, with studies demonstrating improvements in strength, functional capacity, and mobility (Emam et al., 2025; Lim and Goh, 2022). These adaptations likely arise from the combination of metabolic stress, increased motor unit recruitment, and perceptual recalibration that allows older adults to tolerate training stimuli without excessive joint stress. However, the role of perceptual mechanisms in geriatric adaptations remains speculative, as most studies have focused on physiological rather than perceptual outcomes. While direct causal evidence linking perceptual recalibration to improved geriatric outcomes is limited, the observed physiological and functional benefits align with the hierarchical integration model.

### Athletic performance

Athletic applications highlight the versatility of BFR. Systematic reviews demonstrate benefits including improved fatigue management and performance enhancement (Scott et al., 2016). Specific adaptations include increased aerobic capacity and strength (Dong et al., 2025). Practical applications for elite athletes include strategic use during warm-ups, tapering, and recovery (Pignanelli and Burr, 2021). These findings suggest that BFR can modulate effort perception to strategically manipulate training load, allowing athletes to achieve meaningful training stimuli without accumulating mechanical fatigue or increasing injury risk. However, the hypothesis that BFR enhances fatigue resistance through perceptual recalibration remains to be directly tested in longitudinal athletic populations.

### Context-dependent modulation of perceptual responses

Effort perception under BFR is highly context-sensitive. Acute perceptual responses such as RPE and discomfort vary systematically with cuff pressure and exercise modality (Bemben et al., 2015). Hydration status further influences perceptual responses (McCrone et al., 2025), and occlusion pressure contributes to discrepancies between external and internal load (Bielitzki et al., 2024). This variability demonstrates that BFR does not produce stereotyped perceptual responses but instead amplifies the dynamic weighting processes central to the hierarchical integration model, revealing how peripheral, central, and cognitive factors interact to construct the final percept. Future research should determine whether these acute perceptual modulations predict long term behavioral or performance outcomes, and whether they generalize across populations, modalities, and environmental condition.

### Pain management as a clinical application

Pain management represents an important extension of clinical applications. BFR reduces pain and improves function in military personnel with persistent pain (Gray et al., 2025), and meta-analytic evidence supports its use in knee rehabilitation (Chen et al., 2025). Pain reduction under BFR may reflect parallel modulation of nociceptive and interoceptive processing pathways, although the specific mechanisms remain uncertain and may involve processes distinct from those underlying effort perception. These findings suggest that BFR offers a valuable therapeutic approach for individuals with chronic pain or limited load tolerance, although the mechanistic contribution of perceptual recalibration remains to be clarified.

### Summary and integration

BFR reduces pain and effort perception in clinical populations, promotes safe hypertrophy and strength gains in older adults, and supports fatigue management and performance enhancement in athletes. These benefits may contribute to improved functional outcomes across populations, but formal cost-effectiveness analyses and mechanistic studies are needed to determine the extent to which perceptual recalibration contributes to these improvements.

The hierarchical integration model provides a unifying explanation for these diverse applications, linking acute perceptual distortions to chronic adaptations across contexts. These applied findings should therefore be interpreted as consistent with —but not confirmatory of—the multi-timescale adaptation processes described in Chapter 5. Together, they form the conceptual bridge to Chapter 7.

## Toward an integrated hierarchical model of effort perception

### Scope statement

This review aligns with the scope of *Frontiers in Neuroergonomics* by integrating neural, cognitive, and applied perspectives to explain how effort perception is constructed and regulated in real-world movement contexts. By synthesizing peripheral afferent signals, central inhibitory processes, higher-order motor compensation, and cognitive–motivational modulation, the hierarchical integration model provides a neuroergonomic framework for understanding how humans monitor, interpret, and adapt effort during everyday and occupational tasks. The model further highlights how distortions of effort perception under blood flow restriction (BFR) offer a controlled paradigm for probing the neural mechanisms that support performance, fatigue regulation, and adaptive motor behavior in naturalistic settings.

**Figure 1:**
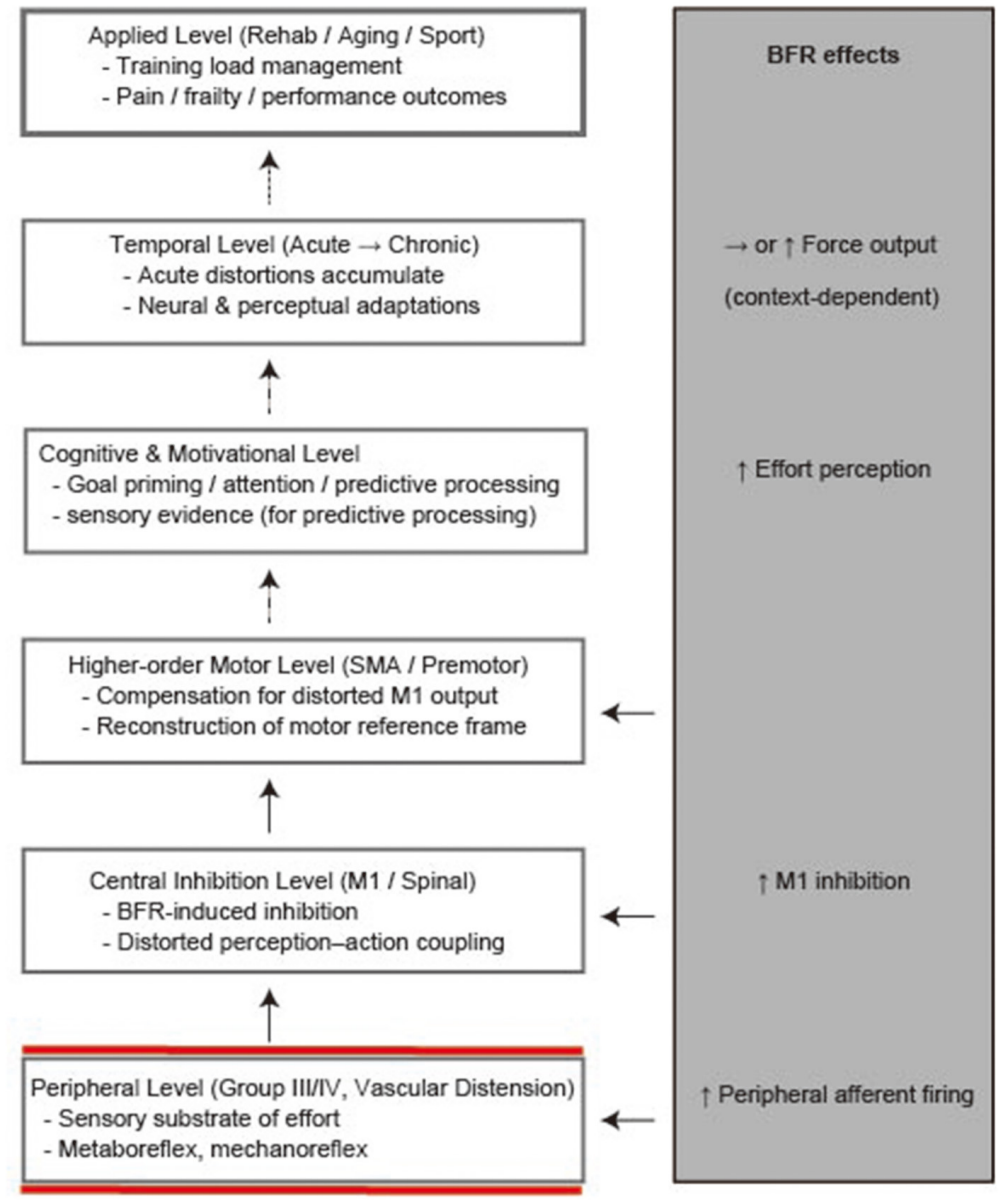
Hierarchical integration model of effort perception under blood flow restriction (BFR). The model comprises six interacting layers arranged from peripheral to applied levels: peripheral afferent input, central inhibition, higher-order motor compensation, cognitive and motivational recalibration, temporal continuity from acute to chronic adaptations, and applied implementation across rehabilitation, aging, and athletic contexts. Bidirectional arrows indicate that changes at one level propagate upward and downward within the hierarchy, reflecting the nested and interactive nature of the system. Line styles indicate evidence strength (solid = strong, dashed = moderate, dotted = speculative). Peripheral and central levels (highlighted red) represent primary sites of BFR-induced perturbation, with secondary influences on higher-order motor and cognitive–motivational processes. The right panel summarizes BFR-induced effects on each layer, including increased peripheral afferent firing, enhanced M1 inhibition, elevated perceived effort, and context-dependent modulation of force output. Predictive coding processes (precision, prediction error, reweighting) operate primarily at the central and cognitive levels and are presented here as a theoretical interpretive framework rather than a validated mechanistic account. Overall, the figure provides a conceptual overview rather than a causal model, organizing current evidence as synthesized in Chapter 7.

The hierarchical integration model summarized in Figure 1 synthesizes peripheral, central, cognitive, temporal, and applied mechanisms into a unified framework of effort perception. Figure 1 illustrates how effort perception emerges from sequential yet interactive layers, with bidirectional arrows representing dynamic feedback and feedforward processes across levels. To avoid overstating the strength of the evidence, the figure encodes evidence strength through line style and color, distinguishing well supported pathways from more speculative links. Building on Chapter 6—which highlighted the clinical, geriatric, and athletic significance of effort regulation—this chapter integrates findings from Chapters 2–6 to articulate a comprehensive hierarchical model. We emphasize that this model organizes existing evidence rather than asserting a fully validated causal structure.

In this model, effort perception is constructed through sequential and interacting layers:

(1) peripheral input from group III/IV afferents and mechanoreceptors;

(2) central inhibition of M1 and spinal circuits;

(3) higher-order compensation by SMA, PMC, and PFC;

(4) cognitive and motivational recalibration;

(5) temporal continuity from acute responses to chronic adaptations;

(6) clinical and applied implementation across populations.

These layers do not operate in isolation; instead, they interact bidirectionally within a nested architecture. Lower-level processes constrain but do not determine higher-level percepts, while higher-level processes modulate the gain, precision, and interpretation of lower-level signals. This bidirectional architecture allows effort perception to be both grounded in physiological reality (bottom-up) and flexibly adapted to context (top-down). To avoid ambiguity, we clarify that “hierarchical” refers to both (a) constraint relations from lower to higher levels and (b) top-down modulation of signal weighting, consistent with predictive-processing accounts.

Peripheral afferents—including group III/IV fibers and vascular distension-sensitive receptors—provide the sensory substrate for effort perception (Haouzi et al., 1999; Kaufman et al., 1988; Mense and Stahnke, 1983). Suppression of the primary motor cortex (M1) distorts the perception–action relationship (Kujirai et al., 1993; Sidhu et al., 2018), while compensatory activity in the supplementary motor area (SMA) and premotor cortex, together with cognitive and motivational processes, reconstructs the perceptual reference frame (Zénon et al., 2015; Schmidt et al., 2012; Takarada and Nozaki, 2018, 2014, 2022, 2024). Chapter 5 demonstrated the continuity from acute responses to chronic adaptations (Laurentino et al., 2012; Centner and Lauber, 2020), and Chapter 6 emphasized the clinical, elderly, and athletic relevance of these processes (Yasuda et al., 2014; Hughes et al., 2017; Scott et al., 2016). This architecture aligns with predictive processing accounts, although the application of predictive coding to effort perception remains a theoretical interpretation rather than a fully validated mechanism.

## Testable predictions generated by the framework

The hierarchical integration model generates several empirically testable predictions regarding how effort perception is reconstructed under blood flow restriction (BFR) and related perturbations. These predictions provide concrete avenues for validating the model's proposed mechanisms across peripheral, central, and cognitive–motivational levels.

1 BFR-induced distortions arise from changes in higher-order cognitive reference frames rather than mechanical load. *Prediction:* Even when mechanical output is held constant, BFR will shift the perceptual reference frame and increase perceived effort. *Empirical test:* Compare effort trajectories under identical load with and without BFR while controlling for mechanical output.

2 The magnitude of M1 inhibition predicts the steepness of effort escalation. *Prediction:* Individuals showing greater BFR-induced suppression of M1 excitability will exhibit a more rapid rise in perceived effort. *Empirical test:* Use paired-pulse TMS to quantify M1 excitability and correlate inhibition magnitude with effort trajectories.

3 Mental fatigue amplifies BFR-induced increases in perceived effort. *Prediction:* Cognitive fatigue will interact with BFR to produce supra-additive increases in effort perception, reflecting shared reliance on higher-order evaluative processes. *Empirical test:* Crossed design manipulating mental fatigue (fatigue vs. control) and BFR (BFR vs. non-BFR) to assess interaction effects on effort ratings.

This hierarchical model extends classical efference-copy accounts (McCloskey et al., 1974; McCloskey, 1981) by demonstrating that efference copy is necessary but insufficient for effort perception. While efference copy provides a prediction of expected sensory consequences, the final percept emerges only after this prediction is integrated with peripheral afferent feedback, modulated by central inhibitory circuits, and recalibrated by cognitive and motivational processes. The model thus preserves the predictive role of efference copy while embedding it within a multi-level architecture. This integration clarifies why effort perception can be preserved even when afferent input is reduced, yet distorted when central inhibitory processes are altered.

Within this integrated perspective, effort perception is a dynamic construct shaped by multiple hierarchical levels. Peripheral inputs provide the raw sensory basis; central inhibition distorts perception–action coupling; higher-order motor regions compensate for these distortions; and cognitive and motivational systems recalibrate reference frames over time. Repeated exposure to distorted effort under BFR leads to chronic adaptations in strength, hypertrophy, and neural plasticity (Cook et al., 2013; Lauber et al., 2021; Frechette et al., 2025). However, the causal contribution of perceptual recalibration to these long-term outcomes remains uncertain, and current evidence supports correlation rather than direct causation. It is important to note that while the hierarchical integration model is supported by converging evidence across levels, direct causal pathways between perceptual recalibration and long-term functional outcomes remain to be empirically established. The model should therefore be understood as an integrative framework that organizes existing evidence and generates testable predictions, rather than as a fully validated causal theory. Nevertheless, the available evidence aligns with the model's multi-level structure (Chen et al., 2025; Gray et al., 2025).

The hierarchical integration model provides a translational framework for designing and optimizing interventions across diverse contexts. In rehabilitation, stabilizing and recalibrating effort perception may improve adherence, motor relearning, and functional recovery (Emam et al., 2025; Lim and Goh, 2022). In athletic and geriatric settings, deliberate manipulation of effort perception can optimize training load, enhance fatigue resistance, and maintain independence (Yasuda et al., 2014; Dong et al., 2025; Pignanelli and Burr, 2021; Ren et al., 2025). Emerging applications in educational and motivational contexts suggest that recalibration of effort perception may facilitate sustained engagement and learning (Scheffel and Gärtner, 2025), although these extensions remain speculative and require empirical validation. Beyond individual outcomes, widespread implementation of BFR-based interventions may contribute to healthcare cost reduction and improved population health, but formal cost-effectiveness analyses are needed before such claims can be substantiated.

Future research should refine and extend the hierarchical integration model by incorporating neuroendocrine responses, autonomic regulation, and explicit cognitive recalibration processes (Pearson and Hussain, 2015; Rossi et al., 2018). Emerging technologies for objective monitoring of effort perception (Bommasamudram et al., 2025) may accelerate translational applications. Neuroimaging studies suggest that condition-dependent cortical reconfiguration may occur across hierarchical levels under BFR and related paradigms, providing preliminary but not definitive support for the integrated framework proposed in this review. Together, these insights underscore the value of a unified, multi-level model capable of explaining both mechanistic foundations and applied outcomes across diverse populations.

The cognitive and motivational layer relies partly on our prior work, and independent replication will be essential to validate these components of the framework.

## Conclusion

This review has synthesized evidence across neurophysiology, psychology, and applied exercise science to propose a hierarchical integration model of effort perception. The originality of this synthesis lies not in identifying isolated mechanisms—most of which were previously known—but in revealing how these mechanisms interact within a unified, multi-level architecture. Three key insights emerge from this integration. First, effort perception is constrained but not determined by peripheral input, as central inhibitory and compensatory processes reshape the perception–action relationship. Second, cognitive and motivational systems recalibrate the reference frame through which effort is interpreted, allowing identical motor outputs to feel more or less effortful depending on context. Third, acute distortions of effort perception accumulate across repeated exposures, although the extent to which these distortions causally contribute to chronic neuromuscular and perceptual adaptations remains to be empirically established. Together, these insights position effort perception as a dynamic construct shaped by interactions across six hierarchical levels.

Evidence synthesized across Chapters 2–7 supports this framework at multiple levels. At the peripheral level, group III/IV afferents and vascular distension-sensitive fibers form the sensory substrate for effort perception (Haouzi et al., 1999; Kaufman et al., 1988). At the central level, suppression of the primary motor cortex (M1) distorts the perception–action relationship, while higher-order motor regions and cognitive processes dynamically reconstruct the perceptual reference frame (Kujirai et al., 1993; Schmidt et al., 2012; Takarada and Nozaki, 2014; Takarada and Nozaki, 2018, 2022). Across timescales, acute distortions accumulate into chronic adaptations in strength, hypertrophy, and neural plasticity (Laurentino et al., 2012; Bemben et al., 2015; Centner and Lauber, 2020). Functional outcomes are demonstrated in clinical, geriatric, and athletic populations, where BFR supports safe hypertrophy, rehabilitation, and performance enhancement (Yasuda et al., 2014; Hughes et al., 2017; Scott et al., 2016). Figure 1 provides a conceptual overview of these relationships while distinguishing well-supported pathways from more speculative links.

This hierarchical model positions effort perception as a dynamic, multilayered system shaped by peripheral afferents, cortical inhibition, higher-order compensation, cognitive recalibration, motivational influences, neuroendocrine responses, and applied contexts. Effort perception is therefore not merely a subjective sensation but a multidimensional phenomenon situated at the intersection of neurophysiology, cognition, and motivation. Its mechanisms have direct implications for human performance, rehabilitation, healthy aging, and broader aspects of goal-directed behavior and self-regulation. The originality of this framework lies in moving beyond the traditional dichotomy between peripheral afferent models (e.g., group III/IV hypothesis) and central corollary-discharge models (e.g., efference-copy hypothesis), demonstrating that both mechanisms operate within a broader architecture in which their relative contributions are dynamically weighted according to context. However, the hierarchical integration model should be interpreted as an organizing framework that generates testable predictions rather than as a fully validated causal theory.

Future research should extend this framework in three directions. First, mechanistic studies should refine understanding of neuroendocrine, autonomic, and cognitive contributions to effort perception (Pearson and Hussain, 2015; Rossi et al., 2018). Second, advances in AI-based analytics and wearable sensors may enable objective, real-time monitoring of effort perception, accelerating translational applications in clinical and athletic settings (Bommasamudram et al., 2025). Third, larger-scale neuroimaging studies are needed to test the hypothesis that BFR induces condition-dependent cortical reconfiguration across hierarchical levels. Such studies will be essential for determining whether perceptual recalibration plays a causal role in long term adaptation or simply co-occurs with other physiological mechanisms. A deeper understanding of effort perception—grounded in multi-level mechanisms and translatable across contexts—will be essential for optimizing human performance, promoting healthy aging, and enhancing rehabilitation outcomes.
